# Effect of LED therapy for the treatment nipple fissures: Study protocol for a randomized controlled trial

**DOI:** 10.1097/MD.0000000000012322

**Published:** 2018-10-12

**Authors:** Thalita Molinos Campos, Maria Aparecida dos Santos Traverzim, Ana Paula Taboada Sobral, Sandra Kalil Bussadori, Kristianne Santos Porta Fernandes, Lara Jansiski Motta, Sergio Makabe

**Affiliations:** a Postgraduate Program in Biophotonics Applied to Health Sciences, Universidade Nove de Julho, UNINOVE; b Hospital Mandaqui, São Paulo, Brazil.

**Keywords:** breastfeeding, healing, mammary diseases, phototherapy, quality of life

## Abstract

**Introduction:**

Poor positioning of the child in relation to the breast and improper suckling are the main causes of nipple fissure. Treatment options for nipple fissures include drug therapy with antifungal and antibiotics, topical applications of lanolin, glycerin gel, creams and lotions, the milk itself, hot compresses, and silicone nipple shields. Studies involving light-emitting diode (LED) therapy have demonstrated anti-inflammatory properties, the enhancement of the wound repair process, and the control of pain. As it does not cause discomfort, is relatively inexpensive and may impede the discontinuation of breastfeeding, phototherapy could be a viable option for the treatment of nipple fissures.

**Aim:**

The principal objective of the proposed study is to evaluate the effectiveness of LED therapy for the treatment of nipple fissures in postpartum mothers.

**Materials and methods:**

One hundred patients treated with a medical diagnosis of bilateral nipple trauma classified as nipple fissures or cracks will participate in the study, randomized into 2 groups: The control group will receive orientation regarding breast care and adequate breastfeeding techniques. The experimental group will receive the same orientation and phototherapy sessions using a device developed especially for the treatment of nipple trauma. Both groups will be followed up for 6 consecutive weeks.

## 1 Introduction

During pregnancy and in the postpartum period, care is fundamental to minimizing problems, such as nipple trauma^[1]^ due to the occurrence of fissures associated with an inflammatory process of the upper layer of the dermis.^[2]^ Around 98% of women can physiologically breastfeed, but many mothers avoid this practice. Nipple fissures are the second major cause of the discontinuation of breastfeeding, followed by the sensation of insufficient milk that many mothers have, leading to the habit of bottle feeding.^[3]^ Nipple fissures are classified as either circular or longitudinal and vary in size. A circular fissure is commonly located at the nipple-areolar junction, whereas a longitudinal fissure is situated throughout the entire length of the nipple either vertically or horizontally, dividing it into 2 halves.^[4]^

Nipple fissures tend to appear in the second or third week of the postpartum period and are a frequent cause of pain that often leads to premature discontinuation of breastfeeding.^[2]^ Poor positioning of the child in relation to the breast, an inadequate frequency or duration of breastfeeding, and improper suckling are the main causes of nipple fissure.^[1]^ Since 1991, both the World Health Organization (WHO) and the United Nations Children's Fund (UNICEF) have dedicated international efforts to protecting, promoting, and supporting exclusive breastfeeding until an infant reaches 6 months of age.^[5]^

The discontinuation of breastfeeding deprives an infant of the essential nutrients, growth factors, and important immunological components in breast milk. For the mother, not breastfeeding hinders uterine involution, increases the risk of postpartum hemorrhage, and increases the risk of ovarian and breast cancer, not to mention the affective aspect of the mother–child bond that is created through the breastfeeding experience.^[6]^

Treatment options for nipple fissures include drug therapy with antifungal agents and antibiotics, topical applications of lanolin, glycerin gel, creams and lotions, the milk itself, hot compresses, silicone nipple shields, and phototherapy.^[7,8]^ It should be stressed that nipple fissures can be a gateway for bacteria, which could lead to more serious conditions, such as abscess and mastitis.^[9,10]^

Phototherapy is the use of electromagnetic waves within the red and infrared spectra that are applied to biological tissues with the aid of low-level light devices, such as light amplification by stimulated emission of radiation (laser) and a light-emitting diode (LED). Studies have demonstrated the anti-inflammatory properties of phototherapy, with the enhancement of the wound repair process^[11–13]^ and the control of pain.^[14,15]^ As it does not cause discomfort, is relatively inexpensive, and may impede the discontinuation of breastfeeding, phototherapy could be a viable option for the treatment of nipple fissures.^[16]^

## 2 Methods/Design

The principal objective of the proposed study is to evaluate the effectiveness of LED therapy for the treatment of nipple fissures in postpartum mothers. The secondary objectives are to evaluate the effect of LED therapy on the healing of nipple fissures in postpartum mothers; evaluate the effect of LED therapy on the control of pain during breastfeeding in postpartum mothers with nipple fissures; and evaluate the impact of the treatment of nipple fissures with LED therapy on the quality of life of postpartum mothers.

**Figure 1:**
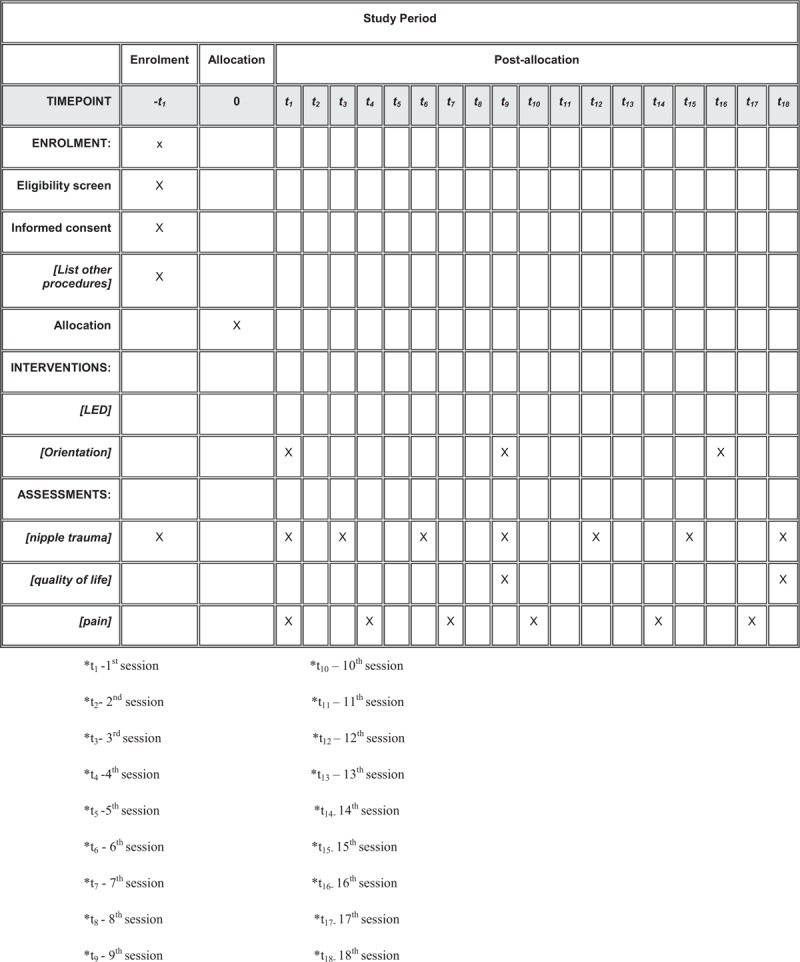
Schedule of enrolment, interventions, and assessments of the study.

This protocol follows the SPIRIT (Standard Protocol Items for Randomized Trials) recommendations displayed in Fig. 1 and Table 1 .

**Table 1:**
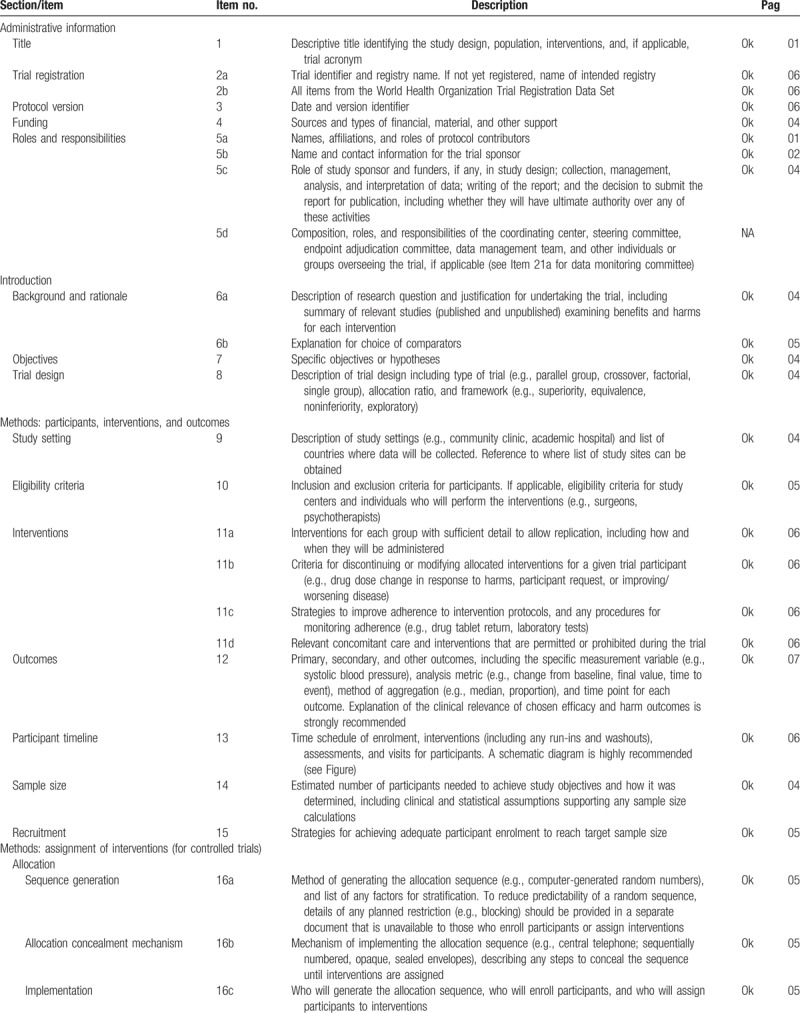
SPIRIT 2013 checklist: recommended items to address in a clinical trial protocol and related documents^∗^.

**Table 1 (Continued):**
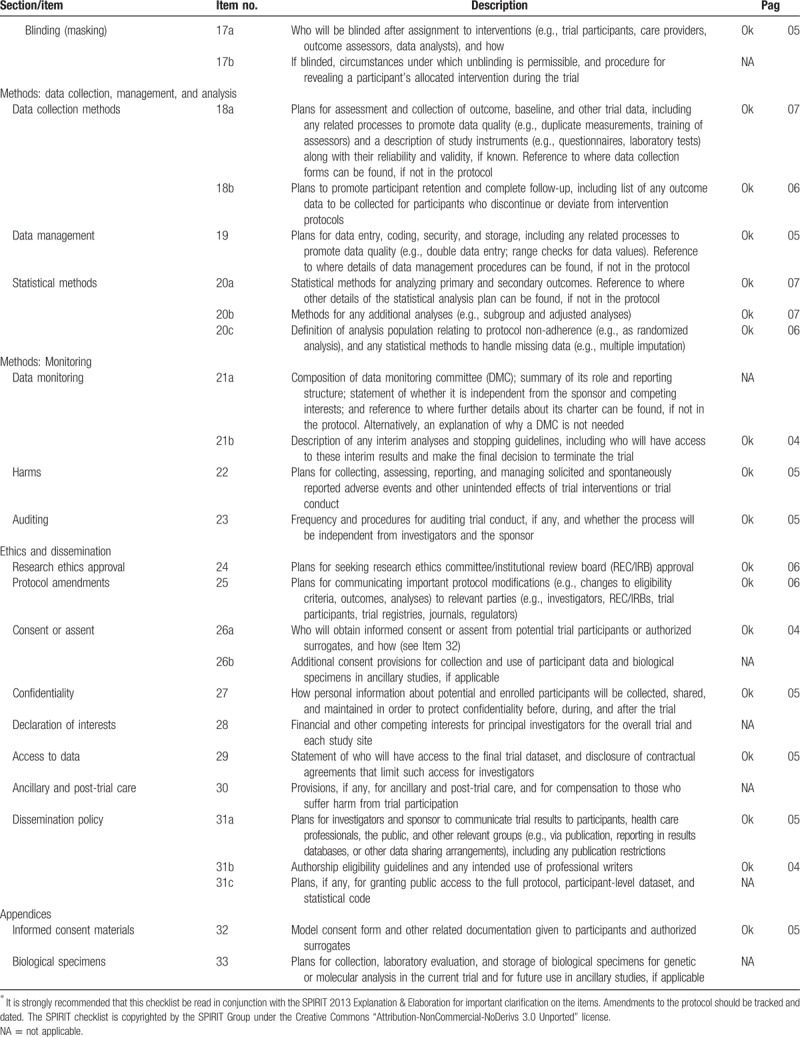
SPIRIT 2013 checklist: recommended items to address in a clinical trial protocol and related documents^∗^.

### 2.1 Methods

A controlled clinical trial will be conducted to evaluate the effects of LED therapy on tissue healing, pain, and quality of life in postpartum mothers with nipple fissures. One hundred patients treated at the Mandaqui Hospital in the city of São Paulo, Brazil, with a medical diagnosis of bilateral nipple trauma classified as nipple fissures or cracks will participate in the study. All patients must sign an informed consent form.

All participants will answer a questionnaire addressing basis information (age, gestation time, and infant's age) and history of nipple fissures, will indicate their pain at baseline using the Visual Analog Scale (VAS), and will answer a quality-of-life questionnaire. All participants will sign a statement of informed consent before the onset of the study.

### 2.2 Eligibility criteria

The following will be the inclusion criteria:

(1) Nursing mothers aged 18 years or older;
(2) Diagnosis of nipple fissure and nipple pain with a minimum score of 1 on the Store and Champion scales;
(3) Having given birth to a healthy, full-term child;
(4) Performing exclusive breastfeeding;
(5) Newborn with no oral, palatal or maxillofacial abnormalities;
(6) Newborn weighing between 2500 and 4000 g.

The following will be the exclusion criteria:

(1) History of psychological disorder;
(2) Presence of mastitis;
(3) Bacterial or fungal infection in breasts;
(4) Use of breast pump or plastic nipple.

The participants will be randomly allocated to either the experimental or control group using a randomization site (http://www.randomization.com). The control group will receive orientation regarding breast care and adequate breastfeeding techniques. The experimental group will receive the same orientation and phototherapy sessions using a device developed especially for the treatment of nipple trauma. Both groups will be followed up for 6 consecutive weeks. The information will be registered in an Excel Program.

The patients will be submitted to an initial evaluation by an examiner blinded to the group allocation. The aim of the evaluation will be to characterize the participant (age, skin color, number of children, schooling, type of birth, previous breastfeeding experience, and time of puerperium) and the nipple [color and type (protruded, semi-protruded, inverted, or hypertrophic)]. After the evaluation, the participants will be sent to begin the interventions.

The patient will be instructed not to use creams, soaps, or any type of ointment on the nipples, to wear bras with wide, firm straps to support the breasts, to remain in a comfortable position during breastfeeding with the infant's body turned completely toward the mother. Secure the breast in a C shape to facilitate the infant's latching, wait for the infant to empty the first breast offered completely before moving to the other breast, and place the tip of the little finger in the corner of the infant's mouth to facilitate its removal from the breast. This information will be given to the women at the initial evaluation as well as in the third and sixth weeks always by the same researcher in both oral and printed (educational brochure) form.^[16]^

The strategy to adherence to the intervention protocol will be information about nipple fissures and complications. All participants, regardless of group, will be part of a support group for the mother and baby with educational activities and care.

### 2.3 Ethical aspects

This study received approval from the human research ethics committee (certificate number: 2.540.438) and is registered with ClinicalTrials (number: NCT03496753).

### 2.4 Intervention

#### 2.4.1 Phototherapy - LED

The following will be the phototherapeutic parameters: total spot area: 1.44 cm^2^; continuous emission mode; output power: 10 mW; infrared wavelength (880–904 nm); fluence: 4 J/cm^2^; and application time: 10 minutes/session. Sessions will be held 3 times a week on alternating days for 6 consecutive weeks, totaling 18 sessions.

#### 2.4.2 Clinical parameters for evaluation of fissures

Nipple fissures will be measured in the first, third, sixth, ninth, 12th, 15th, and 18th sessions using digital calipers (Black Bull) and classified at the beginning and end of the study based on Pereira et al ^[17]^: small (≤ 3 mm), medium (> 3 and ≤ 6 mm), or large (> 6 mm).

#### 2.4.3 Pain scale

Pain will be measured using the Visual Analog Scale in the first, fourth, seventh, 10th, 14th, and 17th sessions by a single researcher. In the experimental group, pain will be measured before the administration of phototherapy.

#### 2.4.4 Quality of life

The impact of nipple fissures on the quality of life of the participants will be evaluated using the self-administered EQ-5D questionnaire in the third and sixth weeks of the study. The EQ-5D is a generic health-related quality-of-life assessment tool developed in Europe that has been translated and validated in different languages, including Portuguese.^[17]^

### 2.5 Outcomes

The main outcomes of the study will be the treatment of nipple fissures and a reduction in nipple pain. The secondary outcomes will be related to the quality of life of the participants.

### 2.6 Sample size

The sample size was calculated based on the results of previous studies^[18]^ and considering a possible dropout rate of 10% to 15% throughout the follow-up period. For a 95% confidence interval, 80% test power, and α = 0.05, it was determined that 50 women would be needed for each group (total: 100 participants).

### 2.7 Statistical analysis

The statistical analysis will be performed with the aid of SPSS 20.0 (IBM Corporation, Armonk, NY). The Kolmogorov–Smirnov test will be used to determine the normality of the data. Depending on the distribution, the data will be expressed as mean and standard deviation for continuous variables. Repeated-measures analysis will be performed considering group and evaluation time. The Mann–Whitney test, *t* test for independent samples, and Fisher exact test will be used for the comparisons, and a *P* value < .05 will be considered indicative of statistical significance. The statistical analysis will be blinded.

## 3 Discussion

This article describes the protocol for a randomized, controlled, clinical trial for the evaluation of the effect of LED therapy for the treatment of nipple fissures in nursing mothers. One of the advantages of the study is the action of LED therapy in the control of nipple pain and the induction of healing of the traumatized breast tissue.^[16]^

Nipple fissures and pain can occur during the lactation period, especially in the initial days of breastfeeding and can even lead to premature weaning. Breast milk is nutritionally ideal for the healthy development of a child and breastfeeding is ideal for orofacial growth and development.^[8,13]^

Nipple pain is generally reported 3 to 6 days after giving birth and can persist for up to 6 weeks.^[1]^ Untreated fissures can compromise the infant's nutrition and can lead to complications, such as mastitis, bleeding, infection, and abscess.^[19]^ The treatments described in the literature include medications, natural extracts, compresses, and lanolin. However, these treatments are not effective with regard to both tissue healing and the control of pain.

Considering the importance of breastfeeding and the high prevalence of nipple fissures, this study proposes a novel way to control nipple pain and accelerate the healing process of nipple fissures through the use of LED therapy, which is a noninvasive technique with no side effects, such as the allergic reactions that can be caused by the ingestion of substances.

## Author contributions

**Methodology:** Thalita Molinos Campos, Maria Aparecida Traverzim, Ana Paula Sobral, Sergio Makabe, Sandra Bussadori, Kristianne Fernandes, Lara Motta.

**Writing – original draft:** Thalita Molinos Campos, Lara Motta.

**Writing – review & editing:** Thalita Molinos Campos, Maria Aparecida Traverzim, Ana Paula Sobral, Sergio Makabe, Sandra Bussadori, Kristianne Fernandes, Lara Motta.
