# Effect of LED therapy for the treatment nipple fissures: Study protocol for a randomized controlled trial: Erratum

**DOI:** 10.1097/MD.0000000000013397

**Published:** 2018-11-16

In the article, “Effect of LED therapy for the treatment nipple fissures: Study protocol for a randomized controlled trial”,^[1]^ which appeared in Volume 97, Issue 41 of *Medicine*, the author order should be Thalita Molinos Campos, Maria Aparecida dos Santos Traverzim, Ana Paula Taboada Sobral, Sergio Makabe, Sandra Kalil Bussadori, Kristianne Santos Porta Fernandes, Lara Jansiski Motta.
